# Top-down pressure and bottom-up responses: a study on provincial Governments’ adoption and textual reproduction of sports industry policies in China

**DOI:** 10.3389/fspor.2025.1665567

**Published:** 2025-11-19

**Authors:** Zhiliang Li, Jingjing Zhou, Jian Liu

**Affiliations:** School of Physical Education, Shenzhen University, Shenzhen, Guangdong, China

**Keywords:** policy diffusion, sports industry policies, policy adoption, policy text reproduction, word embedding

## Abstract

National policy documents, as the starting point of the “document transmission chain”, are often directional and instructive in content. Provincial governments, as transmitters of these policies, possess more precise localized decentralized knowledge and are responsible for their specific implementation. During the downward transmission of policy documents, some provinces tend to directly replicate national policies, which results in provincial governments adopting certain policies, but with unsatisfactory outcomes. Accordingly, this study applies the Word Embedding–Word Mover's Distance method to calculate, for the first time, the variation coefficient of policy text reproduction for provincial governments’ sports industry policies. This method is a semantic distance–based text similarity technique that quantifies the semantic divergence between central and provincial policy texts. The normalized variation coefficient ranges from 0 to 1, where a higher value indicates a greater divergence from the central policy text and a higher degree of local reproduction. On this basis, the study further explores the relationship between the speed of policy adoption and the variation coefficient of policy text reproduction at the provincial level. The results reveal significant heterogeneity among the 31 provincial governments in their policy adoption behaviors, which can be categorized into four types according to the characteristics of adoption speed and variation coefficient: rapid adoption with low variation, rapid adoption with high variation, slow adoption with high variation, and slow adoption with low variation.

## Introduction

Public policy is a fundamental tool of national governance, and policy documents serve as its primary medium. “Governing by documents” is one of the core manifestations of national governance. In China, policy transmission generally involves three levels of government: at the highest level, the central government issues overarching policy documents; at the second level, provincial governments adopt and disseminate the policies issued by the central government; and at the third level, local municipal governments adopt policies issued at the provincial level. In practice, national policy documents, as the starting point of the “document transmission chain”, are often directional, principled, and instructive in nature. Provincial governments, as the transmitters of policies, possess more precise localized decentralized knowledge and are responsible for their specific implementation. The policy diffusion in this study refers to the process by which central government policy influences local government decision-making (1). “Policy adoption” is the act of provincial governments adopting policies issued by higher-level governments (2). In addition to deciding whether and when to adopt a policy, provincial governments may also adapt national policy documents to suit local conditions and create new policies (3). This process of refining and updating the content of policy texts is referred to as “policy text reproduction”. Therefore, policy adoption is not merely a binary state of “presence or absence”, but rather a process characterized by speed, scope, and degree. It is necessary to capture and measure governmental behavior through the substantive changes in policy text content and the actual outcomes of policy implementation, thereby providing theoretical insights into the transmission of sports industry policies between the central and provincial governments.

Research on sports policy diffusion has gradually evolved from asking “whether diffusion occurs” to exploring “how diffusion occurs, to what extent it occurs, and whether it is effective”. In the process of policy diffusion between the central and provincial governments, local governments are influenced not only by the demonstration effects of neighboring regions but also by their response capacity and resource endowments. Existing studies have primarily employed event history analysis, qualitative comparative analysis, and content analysis to reveal the configurational relationships among variables such as external pressure, local resources, and internal learning (4–7). Other scholars have applied machine learning and similarity algorithms to quantify the degree of local governments' content-based responses to central sports policies, providing an empirical foundation for this study (8). However, the existing literature has not focused on the subsector of the sports industry, nor has it measured the effects of further policy text reproduction.

Therefore, this study takes the adoption and text reproduction of sports industry policies by provincial governments as its research focus. By applying the Word Embedding Model (WEM) and the Word Mover's Distance (WMD) algorithm, it measures the variation coefficient (degree of policy text reproduction) of sports industry policies across 31 provincial governments (9). This approach presents a comprehensive picture of provincial policy adoption and text reproduction, marking the first quantitative attempt to measure the textual similarity of sports industry policies between the central and provincial governments. By assessing the degree of provincial governments' policy text reproduction relative to the central government, the study uncovers the underlying mechanisms driving policy refinement and updates, thereby providing empirical evidence to enhance the effectiveness of national sports policy implementation and offering theoretical implications for multi-level sports governance systems worldwide. Based on the above objectives, the research questions of this study are as follows:

1. Do provincial governments adopt central sports industry policies?
2. Why do provincial governments adopt certain policies while rejecting others? Is this related to local development conditions, policy type preferences, or higher-level reward mechanisms? Do they prioritize policies that generate economic benefits?
3. What is the adoption speed and character of provincial governments in relation to policy adoption?
4. To what extent do provincial governments reproduce central sports industry policy texts?
5. What is the relationship between provincial governments’ adoption of central sports industry policies and the variation coefficient of policy text reproduction?

## Literature review

### Central–local relations and the logic of policy implementation

In the early stages, economists primarily focused on the sources of governance efficiency in China. Although centralization is often associated with inefficiency, China has maintained a highly centralized system for a long period while achieving sustained high-speed economic growth (10). The central and local governments differ in the scope of affairs, environments, and powers they face. Some scholars have examined the interaction between central and local governments from the perspective of local governments' policy implementation behaviors (11).

In existing research, studies on local governments' implementation of central policies are mainly divided into two categories: deviations in implementation and adoption behaviors. Regarding the manifestations of implementation deviation, scholars have proposed concepts such as “flexible adaptation”, “nominal–substantive separation”, and “over-implementation” to analyze local governments' deviation behaviors under pressure from higher-level authorities and policies (12, 13). Other scholars have noted that local governments may selectively implement national policies (14).

From the perspective of local governments’ policy adoption behaviors, existing research mainly focuses on how different provinces adopt central policies under political, economic, and social pressures. The adoption behavior of local governments is influenced not only by the goals and directions of central policies but also by the promotion incentives of local officials, the power struggles surrounding local interests, and the overall objectives of regional development (15).

Moreover, the degree of adaptability among provinces in different countries when implementing central policies often varies, largely due to differences in the level of centralization and the discretionary space available to provincial governments in policy execution (16, 17). This phenomenon is particularly evident in both federal and centralized systems. In China, provincial governments frequently reinterpret sports policies through textual revisions, implementation plans, and localized frameworks. While such practices maintain formal compliance with central directives, they allow provinces to adjust policy content according to local realities (18).

### Variations in policy texts during policy diffusion

In recent years, numerous scholars have researched policy diffusion (19–21), particularly focusing on the interactions between central and local governments (22, 23), and further analysing the adoption behaviours of local governments towards central policies (11, 14, 24). However, as the policymaker, the central government faces issues such as information asymmetry and a lack of understanding regarding localized conditions, making it challenging to fully and accurately grasp all the details across different local governments (25). The reproduction of policy documents by provincial governments effectively addresses this limitation.

In the study of policy text reproduction, some scholars consider the adoption speed of governments as an important research variable (11), with a common assumption that the faster the adoption, the better (26). However, Rogers argues that while it is generally advantageous for policy innovations to spread quickly, faster is not always better in the actual process of policy innovation diffusion (21). Therefore, exploring the empirical relationship between policy adoption speed and policy text reproduction can help us reassess the decision-making behaviours of local governments (9, 26).

### Policy research in the field of sports

Content analysis of sports policies has been widely applied as an important tool for understanding policy implementation and its impacts. Henry et al. (27) proposed a comparative analytical framework that classifies and reveals the core themes and trends of sports policies across different countries, emphasizing the value of cross-national comparison in understanding policy differences (27). Christiansen et al. (28) employed content analysis to examine European Union sports policies, finding that they primarily focus on the roles of sports in social inclusion, health promotion, and regional development (28). Wunderlich and Memmert (29) used lexical sentiment analysis to study the dissemination of sports policies on social media, uncovering their public reception and filling a gap left by traditional text-based policy analyses (29).

Research on sports policy texts can generally be divided into two main categories: qualitative manual interpretation and coding of policy texts, and quantitative analysis of policy documents. Methodologically, most studies rely on qualitative tools to manually interpret and code policy texts (30, 31). However, such approaches are often influenced by researchers' subjectivity, making it difficult to achieve precise and scientific quantification. Consequently, some scholars have begun to adopt text mining techniques—such as clustering analysis, co-word analysis, and topic modeling—to investigate policy documents (32–34).

Although some scholars have begun to recognize the importance of comparing policy text similarity (9) and have applied WEM to measure textual similarity (35–37), no studies have yet conducted a systematic and quantitative analysis of variations in sports policy texts between different levels of government using word embedding–based methods. From a methodological perspective, researchers have demonstrated the feasibility of analyzing WEM built on large corpora to more precisely capture semantic shifts in words and reflect broader social changes. They have also utilized distributed word representations to assign multiple low-dimensional vectors to represent different meanings of a single word (38, 39). Although the deeper adoption behaviors of provincial governments are difficult to measure directly, applying WEM to examine the substantive changes in policy content during the process of document transmission can help central authorities better monitor local adoption behaviors and deviations in policy implementation, thereby providing a deeper understanding of the operational dynamics of China's sports industry policies.

### Brief summary

Although the above studies have expanded the theoretical framework of sports policy analysis, systematic research on the diffusion of sports industry policies remains limited. From the perspective of sports policy diffusion, existing studies mainly focus on macro-level aspects such as diffusion directions, patterns and mechanisms. However, they have not delved into the internal textual dimensions of policy diffusion, nor have they measured the degree of variation in sports industry policy texts. In other words, there is still a lack of research assessing how provincial governments specifically implement and reproduce higher-level policies within the sports sector. This study contributes to filling that gap by providing a methodological approach that enables national governments worldwide to quantitatively monitor the implementation processes of subordinate governments, thereby offering a more comprehensive understanding of policy performance and facilitating deeper exploration of the mechanisms underlying policy implementation.

## Materials and methods

### Data sources

The policy samples in this study are collected at two levels: the central government and provincial governments. All data are derived from officially published documents available on government websites.

The study collected all sports industry policy documents publicly released on the central government website between 2010 and 2019, totaling seven documents (see Table 1), which are referred to sequentially as “Central Policy 1–7” according to their release dates. The year 2010 marks the issuance of China's first national policy in the field of the sports industry, while 2019 represents the most recent release to date. Accordingly, the time span for central policy collection is defined as 2010–2019.

**Table 1:** Statistical analysis of sports industry policies issued by the central government.

No.	National policy title	Publication date	Character count
1	Guiding Opinions of the General Office of the State Council on Accelerating the Development of the Sports Industry	24-Mar-10	3,904
2	Several Opinions of the State Council on Accelerating the Development of the Sports Industry and Promoting Sports Consumption	20-Oct-14	6,711
3	Guiding Opinions of the General Office of the State Council on Accelerating the Development of the Fitness and Leisure Industry	28-Oct-16	6,544
4	Opinions of the General Office of the State Council on Further Expanding Consumption in Tourism, Culture, Sports, Health, Elderly Care, Education and Training	28-Nov-16	4,016
5	Guiding Opinions of the General Office of the State Council on Accelerating the Development of the Sports Competition and Performance Industry	21-Dec-18	4,439
6	Notice of the General Office of the State Council on Issuing the Outline for Building a Leading Sports Nation	2-Sep-19	9,021
7	Opinions of the General Office of the State Council on Promoting National Fitness and Sports Consumption to Foster High-Quality Development of the Sports Industry	17-Sep-19	4,926

At the central level, data were obtained from the official website of the Central People's Government of the People's Republic of China (https://www.gov.cn). Only normative documents issued by the Central Committee of the Communist Party of China or the State Council were included, excluding policies independently released by subordinate departments such as the General Administration of Sport of China (https://www.sport.gov.cn). To ensure the authority and comparability of the policy data, the following selection criteria were applied: (1) the issuer must be the State Council or the General Office of the State Council; (2) secondary documents, such as circulars or forwarded ministerial notices, were excluded; and (3) the policy titles were strictly screened to distinguish sports industry policies from general “National Fitness” policies. Ultimately, seven core central sports industry policy documents from 2010 to 2019 were identified as the central-level policy samples.

The local policy samples were obtained from the official websites of 31 provincial-level governments (including provinces, autonomous regions, and municipalities directly under the central government, excluding Hong Kong, Macao, and Taiwan) and their respective sports administrative departments. In total, 141 sports industry policy documents corresponding to the central policies were collected. The identification of local samples was based on a content-matching principle between central and provincial policies: when a provincial policy document explicitly referenced a central policy in its title or main text, it was classified as the adoption text of that specific central policy. Considering the inherent time lag in provincial governments' responses to central directives, the time span for local policy adoption was defined as 2010–2023. All documents were sourced from officially released files published on the websites of provincial governments or their sports bureaus, ensuring the authenticity, legitimacy, and traceability of the dataset.

### Methodology

This study will employ the Word Embedding—Word Mover's Distance method to measure the degree of policy text reproduction, specifically the variation coefficient of policy texts. Traditional methods for calculating the variation coefficient of texts primarily use word-matching algorithms (40) to calculate similarity, and then subtract the similarity from 1 to measure variation. However, these methods struggle to distinguish the semantic and emotional components of vocabulary, and they cannot handle cases where different words convey the same information. By combining the semantic quantification of neural networks in Word Embedding (35) and the measurement of word movement distance in WMD, we can more effectively measure the degree of variation between different policy texts (39, 41).

#### Word embedding

This study is based on the Word2Vec model, utilizing the Skip-gram training approach to predict the context of target words, effectively capturing the contextual relationships between words and training distributed word vectors (42–44). Words with closer contexts are positioned nearer to each other in the vector space. The objective of the Skip-gram model is to maximize the co-occurrence probability of a given target word w*_t_* and its context words w*_(t_* *_+_* *_j)_*, optimizing for higher accuracy in predicting target words and their context, thereby achieving better word representations. The specific objective function can be expressed as:

$$\max\overset{T}{\underset{t=1}{\sum }}\;\underset{-c\leq j\leq c,j\neq 0}{\sum }\;\log P(w_{\left(t+j\right)}|w_{t})$$

Here, **T** represents the total number of words in the training corpus, and **c** denotes the size of the context window (i.e., the number of words observed around the target word). P(w*_(t_* *_+_* *_j)_*∣w*_t_*) is the conditional probability of predicting the context word *w_(t_* *_+_* *_j)_* given the target word *w_t_*. The probability of each context word occurring is calculated using the softmax function:

$$P(w_{\left(t+j\right)}|w_{t})=\frac{exp\;(\;V_{wt+j\;}.V_{wt})}{\sum_{w^{\prime }\in V}\;exp\;(\;V_{w^{\prime }}\cdot V_{wt})}$$

Here, V*_wt_* represents the word vector of the word W*_t_*; **V** is the size of the vocabulary; and $exp\;(V_{w^{\prime }}.\;V_{wt})$ denotes the similarity score between the context word *w_(t_* *_+_* *_j)_* and the target word W*_t_*. This model allows us to generate a fixed-dimensional vector representation for each word, where semantically similar words are positioned closer together in the vector space. To improve the computational accuracy of our model, we fine-tuned a pre-trained Word2Vec model (45). In simple terms, Word Embedding is implemented using the Word2Vec tool, which is trained with the Skip-gram approach.

#### Word Mover's distance

After obtaining the vector representation of each word, this study uses the Word Mover's Distance algorithm to calculate the semantic distance between policy texts. WMD calculates how words from one text can be “moved” to match words in another text with minimal effort (46, 47). The core idea of WMD is to measure text variation by calculating the “moving cost” of words between two documents (48). In this study, the seven sports industry policies issued by the central government are used as baseline texts, and each of the 31 provincial government policy texts is compared with these seven central policies. The 141 provincial government policy texts are reproductions based on national policies, and each provincial policy needs to be compared to the corresponding national policy to calculate similarity. The seven central policies are denoted as *P*_1_, *P*_2_, …… , *P*_7_; the 141 reproduced provincial policies are denoted as *R*_11_, *R*_12_, …… , *R*_71_, *R*_72_, …… , *R*_141_, where *R_ij_* represents the reproduced policy of the j-th provincial government corresponding to the i-th national policy. The similarity between each *R_ij_* and its corresponding national policy *P_i_* is calculated using the following formula:

$$\mathrm{WMD}(P_{i},R_{ij})=min\underset{k,l}{\sum }\;T_{kl}\cdot d(V_{P_{ik}},V_{R_{ijl}})$$

Here, $V_{P_{ik}}$ is the word vector for the word $w_{P_{ik}}$ in the national policy P*_i_*; $V_{R_{ijl}}$ is the word vector for the word $w_{R_{ijl}}$ in the provincial policy R*_ij_*. T*_kl_* represents the optimal “transportation plan” from words in the central policy to words in the provincial policy, indicating the movement allocation of each word from the central to the provincial text. $d(V_{P_{ik}},V_{R_{ijl}})\;$ denotes the Euclidean distance between $w_{P_{ik}}$ and $w_{R_{ijl}}$.

Through the above computation, a similarity matrix is obtained, consisting of 7 columns (representing the seven national policies) and 141 rows (representing the provincial governments' reproduced policies), forming a 7 × 141 similarity matrix. Each element represents the semantic distance between a pair of policy texts. Let the semantic distance between the *i^th^* national policy and the *j^th^* provincial policy be defined as:

$$D_{ij}=\mathrm{WMD}(P_{i},R_{ij})$$

To eliminate the influence of differences in text length and to achieve dimensional consistency, this study applies a min–max normalization to *D_ij_*, obtaining the standardized distance as follows:

$$V_{ij}=\frac{D_{ij}-\min(D_{i})}{\max(D_{i})-\min(D_{i})}\in [0,1]$$

Here, *V_ij_* reflects the degree of content variation of the *j^th^* provincial policy relative to the *i^th^* national policy. A larger value indicates a greater semantic difference between the provincial and central texts, representing a higher level of policy text reproduction.

On this basis, the study takes the average variation value of each province across all national policies as the province's overall Policy Text Reproduction Variation Coefficient:

$$VC_{p}=\frac{1}{K}\overset{K}{\underset{i=1}{\sum }}\;V_{i,p}$$

Here, *K* represents the total number of national policies. The variation coefficient VC*_p_* ranges from 0 to 1, where a larger value indicates that the reproduced provincial text differs more significantly from the central text, reflecting a higher degree of innovation.

Based on the above method, seven national policies and 141 provincial policy texts were input into the Word2Vec model to generate word embedding vectors for each text. Subsequently, the WMD algorithm was applied to calculate the semantic distance between each national policy and its corresponding provincial policy text, thereby measuring the variation degree of provincial governments' policy text reproduction.

In the WMD algorithm, the concept of distance refers to how far the word vectors in one text must “move” to optimally match the word vectors in another text; essentially, it represents a semantic-space-based “transportation cost” (35). Therefore, a larger WMD value indicates greater semantic divergence between the two texts. After standardizing this distance, it is denoted as *V_ij_*, and the average variation coefficient *VC_p_* across all national policies for each province is further calculated to reflect the overall level of divergence in provincial policy text reproduction.

### Research design

The research approach is as follows: First, data collection and analysis. Collect sports industry policy texts issued by both the central and provincial governments, filter out policies directly related to the sports industry, and analyze the adoption of all central sports industry policies by the 31 provinces. Second, for provinces that adopt central policies, further analyze the adoption speed and the character count of the adopted texts. Third, conduct text cleaning and tokenization. Decompose the policy texts into individual words, remove irrelevant information (such as stop words, punctuation, etc.), and perform word embedding. Fourth, calculate the WMD to establish a vertical variation coefficient of policy content between central sports industry policies and those issued by the 31 provincial governments, thereby quantifying the degree of policy text reproduction. Fifth, Analyzing the Relationship between Provincial Governments' Policy Adoption Speed and Policy Text Reproduction.

## Results

### Policy adoption

This study conducts a comprehensive analysis of the policy adoption behaviours of provincial governments by systematically examining the adoption of seven central policies across different provinces. The analysis addresses questions such as whether provincial policies adopt central policies, the speed of adoption, and the character count of adopted policies.

#### Policy adoption rate

Table 2 presents an overview of the adoption of seven central sports industry policies by 31 provincial governments from 2010 to 2023. The table includes the dates of policy releases, with blank cells indicating missing policy adoption. The results show that provincial governments generally follow central policies as guiding principles, with all policies being released later than those of the central government. Only Hubei province demonstrated an instance of policy piloting in the field of sports industry. Additionally, different provinces exhibit varying time lags and levels of adoption. From the perspective of policy adoption, while provincial governments demonstrate a high level of responsiveness to central policies, some provinces have not adopted or have delayed adoption of certain policies. In terms of adoption timing, the adoption of central policies by provincial governments tends to be concentrated around similar periods following the release of central policies, suggesting a horizontal effect of learning and imitation among governments.

**Table 2:** Overall adoption status of 31 provincial governments.

Provinces	Central policy 1	Central policy 2	Central policy 3	Central policy 4	Central policy 5	Central policy 6	Central policy 7
	24-Mar-10	20-Oct-14	28-Oct-16	28-Nov-16	21-Dec-18	02-Sep-19	17-Sep-19
Beijing	15-May-12	14-Jul-15				27-Nov-20	11-Jun-21
Tianjin		29-Jul-15	12-Jul-17	28-Feb-17		08-Jan-20	19-Nov-20
Hebei	17-Jan-11	29-May-15	16-Jun-17	17-Jul-17	28-May-19		30-Apr-20
Shanxi	12-Aug-11	01-Aug-15	16-Aug-17	30-Mar-17			18-Feb-21
Inner Mongolia		30-Sep-15	28-Jun-17	14-Jul-17	26-May-23		11-May-22
Liaoning		16-Aug-15	21-Dec-16	30-Sep-17		18-Jan-22	
Jilin		08-Dec-15	25-Jan-19	20-Jun-17		01-Jun-20	
Heilong jiang	12-Jun-12	25-Aug-15	06-Mar-19	24-Jul-17		24-Sep-20	
Shanghai	20-Apr-12	01-Jul-15					
Jiangsu	10-Sep-10	09-Jun-15	17-May-17	15-May-17		30-Jun-20	
Zhejiang	03-Aug-10	25-Jun-15	18-Dec-17			23-Mar-21	30-Apr-20
Anhui	21-Sep-11	25-Jun-15	07-Feb-17	26-May-17		17-Sep-21	
Fujian	15-Mar-11	18-Aug-15	17-Oct-17	11-Aug-17			
Jiangxi		05-Aug-15	25-Jan-18	27-Apr-17	15-Jan-20	29-Jun-21	28-Aug-20
Shandong	17-Sep-12	28-Aug-15		19-May-17		10-Dec-21	17-Dec-21
Henan	12-Feb-11	29-Jul-15				09-Dec-21	08-Jan-21
Hubei		07-Aug-15	30-Apr-17			26-Dec-17	08-Jul-20
Hunan	31-Jul-14	09-Oct-15	12-Jul-17	28-Jun-17		12-Jul-20	
Guangdong	26-Dec-12	10-Aug-15	31-May-17	01-Jun-17		10-Sep-20	
Guangxi	15-Dec-11	29-Jul-15	08-Feb-18			17-Jun-20	
Hainan		07-Aug-15	14-Sep-17	31-Jan-18			
Chongqing		24-Jun-15	28-Apr-17			01-Mar-21	
Sichuan	12-Oct-12	11-Jul-15	01-Aug-17	01-Jul-17		21-Oct-22	28-Jul-20
Guizhou		17-Aug-15	13-Mar-17	01-Dec-17		22-Jun-21	
Yunnan	11-Mar-11	08-Jun-15	05-Jul-18	24-May-17	14-Jun-19	11-Aug-20	07-May-20
Tibet		08-Aug-15	02-Aug-18		10-Jul-20	25-Nov-20	29-Jan-21
Shaanxi	17-Dec-10	29-May-15	16-May-17	13-Mar-18		05-Mar-20	
Gansu	19-Oct-12	28-Jan-15	06-Aug-18	15-Jul-17	20-Apr-20	25-Mar-20	
Qinghai		04-Jun-15	24-Oct-17	09-Jun-17	16-Aug-19	09-Jun-20	
Ningxia		17-Jul-15	01-Feb-17				
Xinjiang		15-Sep-15	18-Apr-19				09-Oct-20

**Figure 1:**
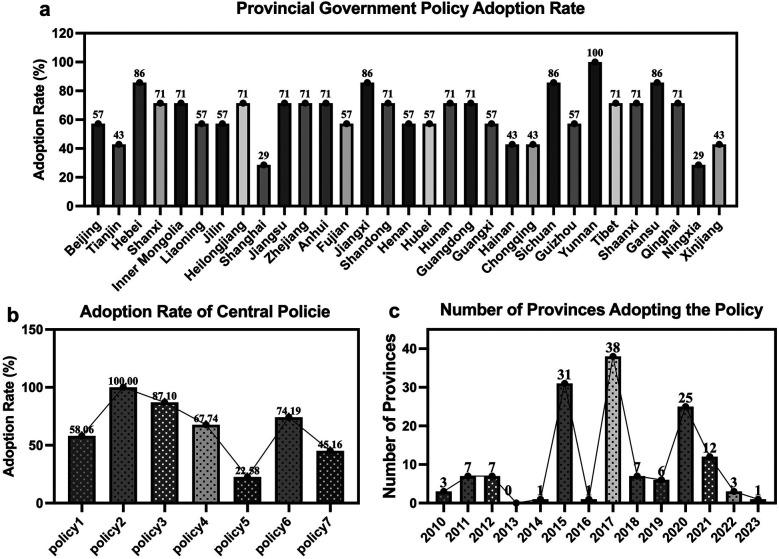
Policy adoption rates and number of provinces adopting policies. Legend for **(a)** adoption rates of 31 provincial governments **(b)** adoption rates of the 7 central policies **(c)** number of provinces adopting central sports industry policies annually from 2010 to 2023.

Figure 1 below presents the policy adoption rates of the 31 provincial governments, the adoption rates of central policies, and the number of provinces adopting central policies from 2010 to 2023. In Figure 1a, there are noticeable differences in policy adoption rates among provinces. Yunnan has the highest adoption rate, reaching 100%, followed by Hebei, Jiangxi, and Gansu, each with an adoption rate of 86%. In contrast, Shanghai and Ningxia exhibit relatively low adoption rates, at 29%. These differences are related to factors such as national policy requirements, the emphasis local governments place on the sports industry, and local economic conditions.

In Figure 1b, Central Policy 2 was adopted by all 31 provinces, and Central Policy 3 also shows a high adoption rate. In contrast, Central Policy 5 was adopted by only 22.58% of the provinces. Factors such as the goal orientation of the policy, implementation difficulty, and economic and social benefits greatly contribute to the variation in central policy adoption rates.

Figure 1c shows that the number of provinces engaging in policy adoption peaked in 2015, 2017, and 2020, with 31, 38, and 25 provinces, respectively. After 2020, the number of provinces adopting policies has shown a significant downward trend. The study observes that there were troughs in policy adoption by provincial governments in 2013, 2014, and 2016. Since 2010 marks the release of China's first sports industry policy, there is a lag in policy adoption by provincial governments. Additionally, 2019 is the year of the last sports industry policy issued by the central government, and by 2023, the peak period for policy implementation and adoption has passed. Therefore, when analyzing the number of provinces adopting policies, we should disregard the data from these two years to avoid skewing the chart's interpretation. The fluctuations in Figure 1c mainly reflect periods of intensive policy releases or key promotion phases by the central government. Furthermore, peaks in adoption were concentrated among provincial governments in certain years.

#### Policy adoption speed

**Figure 2:**
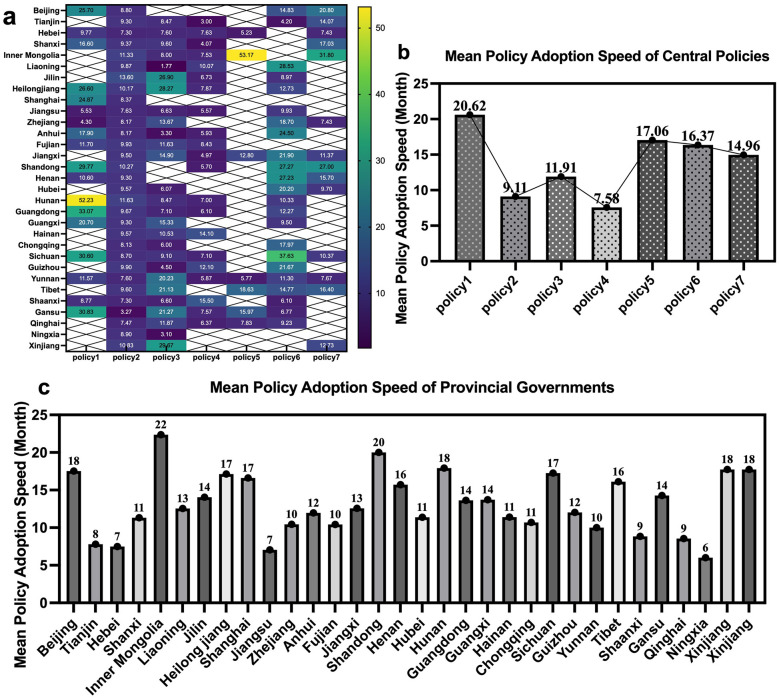
Adoption speed of provincial and central policies. Legend for **(a)** distribution of policy adoption speed by 31 provincial governments **(b)** average adoption speed of the 7 central policies **(c)** average adoption speed of policies by 31 provincial governments.

Figure 2 illustrates the adoption speed of central sports industry policies by provincial governments, measured in months. A higher value indicates a longer adoption time and a slower adoption speed. Figure 2a shows the overall adoption speed of the seven central sports industry policies by the 31 provincial governments. Lighter colours indicate a longer time required for provincial governments to adopt the policies, and vice versa. From the perspective of regional differences, the developed eastern regions, such as Beijing, Shanghai, and Tianjin, generally display more dark-coloured areas, indicating a faster adoption speed. These developed areas have advantages in resource allocation, administrative efficiency, and policy implementation capacity, enabling them to respond quickly to central policy directives. In contrast, western regions such as Yunnan, Shaanxi, Gansu, and Qinghai also show dark colours for multiple central policies. Although these areas have relatively slower economic development, they have similarly demonstrated a rapid adoption speed.

From the perspective of central policies (Figure 2a), Policy 2 and Policy 4 show an overall trend of faster adoption speeds, with regions like Gansu and Tianjin displaying the darkest colours, indicating adoption within just over three months. The rapid adoption speed of Central Policy 2 can be attributed to its significant role in promoting the development of China's sports industry. Released in 2014, the “Several Opinions of the State Council on Accelerating the Development of the Sports Industry and Promoting Sports Consumption” (Policy 2) not only established 11 statistical categories for the sports industry in 2015 but also positioned the sports industry as a key factor in driving national consumption. In contrast, Policies 1 and 5 exhibit slower overall adoption speeds, with extreme values observed in Inner Mongolia and Hunan at 53.14 and 52.23 months, respectively. This reflects the adaptability and complexity of these central policies. Notably, for Central Policy 3, Liaoning took only 1.77 months to adopt the policy, indicating a high level of feasibility and applicability of this policy in the region.

Figure 2b shows the average adoption speed of the seven central sports industry policies. Among them, Policy 1 has the slowest average adoption speed, taking 20.62 months, while Policy 4 (“Opinions of the General Office of the State Council on Further Expanding Consumption in Tourism, Culture, Sports, Health, Elderly Care, Education and Training”) has the fastest adoption speed, at only 7.58 months.

Figure 2c illustrates the distribution of the average policy adoption speed among the 31 provincial governments. The results show that Inner Mongolia and Shandong have the longest average adoption times, requiring approximately 22 and 20 months, respectively, to adopt central policies. In contrast, Ningxia, Hebei, and Jiangsu exhibit the shortest adoption times, averaging only 6–7 months. However, the majority of provinces display an average adoption speed ranging between 10 and 18 months, indicating a relatively stable response pace among provincial governments during the policy adoption process.

#### Policy adoption characters

**Figure 3:**
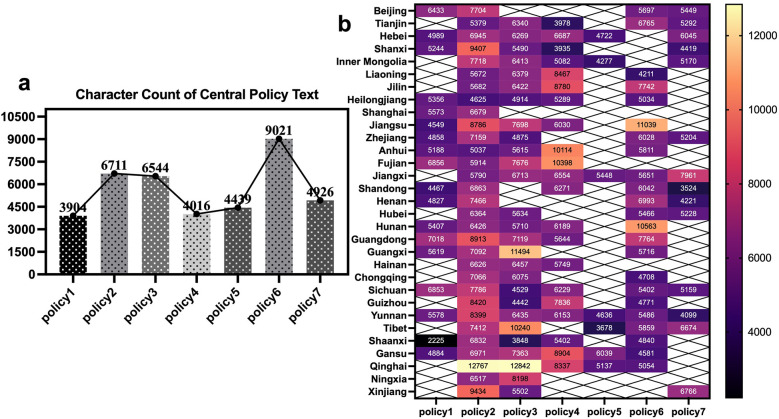
Character counts of policy adoptions. Legend for **(a)** character counts of the 7 central sports industry policy texts **(b)** distribution of character counts in policy adoptions by 31 provincial governments.

Figure 3 shows the character counts of the seven central sports industry policy texts and the character counts of the policies adopted by the 31 provincial governments. Figure 3a indicates that Central Policy 6, issued in 2019, has the highest character count, reaching 9,021 characters. This is because the “Notice of the General Office of the State Council on Issuing the Outline for Building a Leading Sports Nation” treats the sports industry as a major project, covering various dimensions of sports industry development, making it the most detailed and complex policy in terms of content.

In contrast, Central Policy 1 (Guiding Opinions of the General Office of the State Council on Accelerating the Development of the Sports Industry), issued in 2010, has the fewest characters, with only 3,904, and it also has the slowest adoption speed among all policies. As the initial guiding document for China's sports industry, this policy is relatively concise, providing only a general framework without specific implementation details for provincial governments. Consequently, the adoption speed of Central Policy 1 by provincial governments is slower, with significant character count variations. However, Central Policy 4 has the fastest adoption rate and one of the lowest character counts. This suggests that when the character count of a central policy is low, the adoption behaviour of local governments tends to be polarized.

Figure 3b presents the overall character counts of provincial governments' adopted sports industry policy texts compared with the corresponding central policies. Provinces such as Qinghai, Fujian, Jiangsu, and Guangxi exhibit relatively high character counts, exceeding 10,000 characters in certain policies—particularly Qinghai, where the reproduced texts for Policy 2 and Policy 3 are nearly twice as long as the corresponding central policies. In contrast, provinces such as Shaanxi, Shandong, and Tibet display comparatively shorter adopted texts, with character counts below those of the central policies. However, a smaller number of characters in these provincial adoptions does not necessarily imply weaker policy implementation effectiveness.

### Policy text reproduction

The study uses a matrix of variation coefficients for the reproduced policy texts of provincial governments to understand the degree of text reproduction variation after adopting central policies. Table 3 shows that there are significant differences in variation coefficients across different policies and provinces, with most provinces’ variation coefficients concentrated between 0.3 and 0.4. On one hand, Shaanxi exhibits the highest variation in Central Policy 1, with a coefficient of 0.6427, the maximum value among all data points. On the other hand, Anhui shows the lowest variation in Central Policy 4, with a coefficient of only 0.1823, the minimum value among all data points, indicating that Anhui almost fully retained the content of the central policy.

**Table 3:** Variation coefficient matrix of policy text reproduction for 31 provincial governments.

Provinces	Central policy 1	Central policy 2	Central policy 3	Central policy 4	Central policy 5	Central policy 6	Central policy 7
Beijing	0.4382	0.3751				0.4656	0.4715
Tianjin		0.3923	0.3442	0.5551		0.3848	0.4325
Hebei	0.2756	0.2651	0.3185	0.4280	0.4303		0.4390
Shanxi	0.4261	0.2420	0.3177	0.2363			0.5558
Inner Mongolia		0.2476	0.3339	0.3434	0.4366		0.4360
Liaoning		0.2979	0.3114	0.4949		0.4169	
Jilin		0.3479	0.3993	0.4731		0.3648	
Heilong jiang	0.3727	0.4257	0.3845	0.3434		0.4325	
Shanghai	0.4284	0.3045					
Jiangsu	0.4132	0.3191	0.3132	0.5050		0.3949	
Zhejiang	0.3668	0.2834	0.3329			0.4601	0.4430
Anhui	0.3602	0.3004	0.1823	0.4316		0.4741	
Fujian	0.4959	0.4505	0.4263	0.4567			
Jiangxi		0.2772	0.4182	0.4010	0.3950	0.3372	0.4026
Shandong	0.3638	0.3269		0.5009		0.4392	0.5197
Henan	0.2868	0.2534				0.4431	0.3638
Hubei		0.2855	0.4036			0.4172	0.4397
Hunan	0.4070	0.3301	0.4121	0.5124		0.4040	
Guangdong	0.4386	0.3552	0.3713	0.4842		0.4213	
Guangxi	0.4296	0.3156	0.3352			0.4085	
Hainan		0.3377	0.4144	0.5666			
Chongqing		0.3117	0.3374			0.3770	
Sichuan	0.3663	0.3236	0.3880	0.4864		0.4570	0.4267
Guizhou		0.3939	0.4675	0.4721		0.3895	
Yunnan	0.3668	0.2954	0.4397	0.4128	0.3508	0.3896	0.4120
Tibet		0.3526	0.3970		0.4866	0.4510	0.3546
Shaanxi	0.6427	0.2752	0.4156	0.4713		0.4099	
Gansu	0.4488	0.3122	0.4707	0.4380	0.4564	0.4762	
Qinghai		0.3426	0.5359	0.4724	0.4774	0.3822	
Ningxia		0.4026	0.3166				
Xinjiang		0.3465	0.2818				0.3225
Mean	0.4071	0.3255	0.3729	0.4517	0.4333	0.4172	0.4300

From the average variation coefficient of central policies, Policy 4 has the highest average, reaching 0.4517, and it was also adopted at the fastest rate. Notably, Central Policy 2 from 2014 (“Several Opinions of the State Council on Accelerating the Development of the Sports Industry and Promoting Sports Consumption”) has an adoption rate of 100% and is considered a milestone policy in the development of China's sports industry. It has the lowest variation in policy text reproduction among provincial governments, with a coefficient of only 0.3255. This indicates that provincial governments maintained a high level of consistency with the central government in this policy, which suggests strong policy enforcement rigidity or clearly defined guidelines. In summary, this matrix provides important empirical evidence for further research on the mechanisms of policy text reproduction by provincial governments.

**Figure 4:**
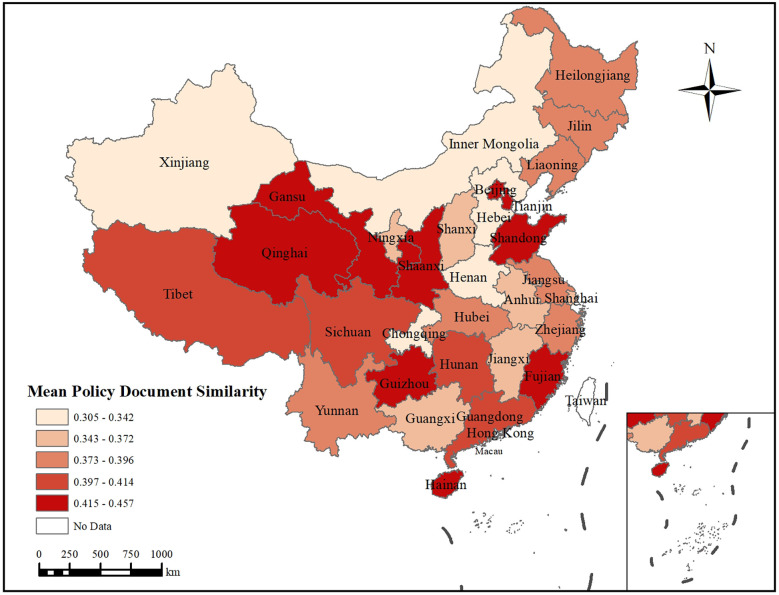
Spatial distribution of average variation coefficients. This map is based on the standard map with the review number GS (2024) 0,650 downloaded from the standard map service website of the ministry of natural resources. The base map has not been modified.

Figure 4 presents the average variation coefficients of policy text reproduction for the 31 provincial governments. The study divides the variation coefficients of each province's policy text reproduction into several intervals, with different colours representing the ranges of average variation coefficients. The range of average variation coefficients spans from 0.305 to 0.457, with darker colours indicating larger values and, consequently, a greater degree of policy text reproduction variation.

First, from a macroscopic perspective, the variation coefficient shows a gradual increase from the eastern coastal regions to the western inland regions. Provinces such as Qinghai, Gansu, and Guizhou in western China exhibit relatively high average variation coefficients (0.415–0.457), indicating that policies in these regions must balance local cultural adaptation and administrative complexity. Second, Gansu and Qinghai, Tibet and Sichuan, Hunan and Guangdong, as well as Beijing and Tianjin, display similar variation coefficients among geographically adjacent provinces. This suggests that provincial governments tend to reference and learn from neighboring regions when reproducing policy texts. Third, Beijing, Tianjin, Fujian, and Hainan, all located in coastal areas, also exhibit relatively high average variation coefficients (0.415–0.457).

### Analysis of the relationship between the speed of policy adoption and the variation coefficient of central-local sports industry policy diffusion

**Figure 5:**
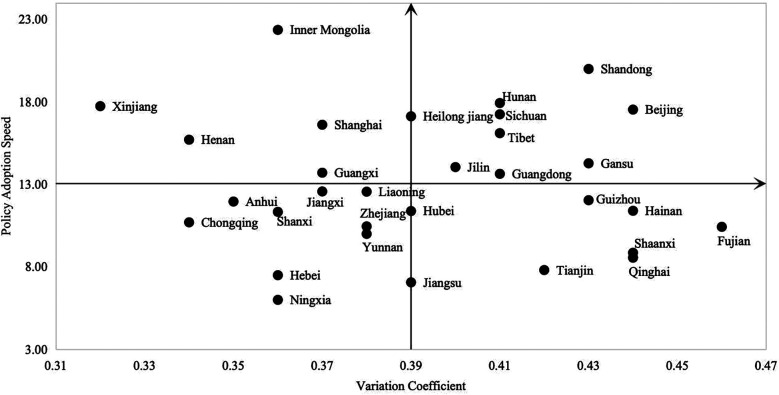
Scatter distribution of policy adoption speed and variation coefficients of sports industry policies among provincial governments.

As analyzed above, the adoption of sports industry policies by provincial governments varies not only in timing but also in the degree of policy text reproduction. This study constructs a two-dimensional coordinate system, with the average policy adoption time of 31 provincial governments on the vertical axis and the average degree of policy text reproduction on the horizontal axis. The data for each province are plotted as scatter points, and based on their distribution characteristics, four distinct policy behavior combinations are identified (Figure 5).

The first type is the “Rapid Adoption + Low Variation” type, represented by Chongqing, Ningxia, Hebei, and Shanxi. The average adoption time is 8.08 months, and the average variation coefficient is 0.37. These provinces typically have a strong policy implementation system and resource allocation capacity, enabling them to respond quickly to central policy requirements. The extent of text reproduction is relatively small, reflecting a high degree of policy consistency and implementation homogeneity.

The second type is the “Rapid Adoption + High Variation” type, including Fujian, Hainan and Qinghai. The average adoption time is 8.90 months, and the variation coefficient reaches 0.42. These provinces also respond to central policies promptly, but actively make local adjustments during the adoption process, emphasizing the adaptability and contextuality of policy content. This represents a typical example of “rapid response + proactive adaptation” by local governments.

The third type is the “Slow Adoption + High Variation” type, represented by Shandong, Tibet, Sichuan, and Beijing. The average adoption time is 15.76 months, and the variation coefficient is 0.43. Local responses are relatively delayed after the release of central policies, and during subsequent adoption, these provinces demonstrate higher autonomy and text reconstruction ability.

The fourth type is the “Slow Adoption + Low Variation” type, mainly including Xinjiang, Inner Mongolia, and Henan. The average adoption time is 19.17 months, and the variation coefficient is 0.34. This type exhibits a dual characteristic of “delayed adoption + limited adjustment”. While local governments delay their response to central policies, the extent of text adjustments is relatively small, indicating weaker implementation willingness or a high degree of reliance on central policies.

Overall, local governments exhibit different strategic combinations in the policy adoption and reproduction process. Although there is no single linear relationship between adoption speed and the variation coefficient, their coupling dynamics reflect differentiated response mechanisms under the pressures of institutional constraints, resource capacity, and adaptation needs.

## Discussion

This study aims to reveal the current patterns and underlying mechanisms of sports industry policy diffusion in China within the central–local government framework, focusing on the policy adoption behaviors and textual reproduction characteristics of provincial governments during the transmission of central policies. The findings indicate that while local governments generally demonstrate a high level of responsiveness in policy adoption, there are significant differences in adoption rate, adoption speed, and policy text length. Developed regions tend to adopt policies more rapidly, whereas some central and western provinces exhibit delayed or selective adoption. At the level of policy text reproduction, the variation coefficients of provincial policies range mainly between 0.30 and 0.45, showing a spatial pattern of “low in the east and high in the west” with regional convergence among neighboring provinces.

Furthermore, the coupled analysis reveals that there is no simple linear relationship between policy adoption speed and the variation coefficient of policy text reproduction. The 31 provinces can be categorized into four typical response types: *rapid adoption with low variation*, *rapid adoption with high variation*, *slow adoption with high variation*, and *slow adoption with low variation*. These types illustrate the diverse diffusion logics of local governments shaped by institutional pressures, resource capacities, and policy adaptation needs.

### Overall characteristics of provincial Governments’ policy adoption in the sports industry

This study finds that between 2010 and 2023, provincial governments exhibited significant differences in adopting central sports industry policies. Although most provinces showed a high level of responsiveness to central policies, some provinces still displayed delayed or non-adoption of certain policies.

For example, Yunnan's policy adoption rate reached 100%, while the adoption rates in Shanghai and Ningxia were relatively low, at 29% each. As a leading economic municipality, Shanghai prioritizes the development of innovative and high-tech industries. The sports industry in Shanghai has strong participation from private capital and is largely market-driven, giving the city greater independence and autonomy in policy formulation.

Conversely, Yunnan's 100% adoption rate reflects the local government's recent efforts to vigorously promote the sports sector as an important means to boost the local economy and attract tourism. This demonstrates the concept of “selective adaptation” in policy diffusion theory, where local governments decide whether to adopt a policy based on its applicability and resource costs (11).

In 2019, Hubei Province took the initiative to implement Policy 6 ahead of the central government, demonstrating an instance of policy experimentation. This phenomenon provides a unique perspective on the proactive role of local governments in policy innovation (49). Traditional policy diffusion theory views central-to-local policy diffusion as a top-down process, with local governments responding to directives from the central government (50). However, Hubei's proactive experimentation suggests that local governments have an active role in policy innovation within certain fields. This “bottom-up” diffusion model is essentially an attention-driven process of policy diffusion: “local attraction generation” — “performance reporting” — “higher-level attention arousal” — “policy adoption by higher authorities” — “policy dissemination by higher authorities” (51). For the promotion of local officials, the process of “local innovation — higher-level adoption and dissemination — subsequent adoption by other regions” serves as a motivating factor.

Central Policy 2, issued in 2014, achieved a 100% adoption rate across the 31 provinces, with rapid adoption speeds and low variation. This policy positioned the sports industry as a key strategy for promoting economic growth, expanding domestic demand, and enhancing public health. This clear macroeconomic objective led local governments to view it as an opportunity to drive local economic and social development, prompting them to actively adopt the policy to reap its benefits. Central Policy 4, issued in 2016, exhibited similar characteristics. Therefore, the study concludes that policies with significant economic incentives tend to accelerate the adoption speed by local governments (52–54).

### Analysis of the relationship between policy adoption speed and textual reproduction

In the diffusion process of sports industry policies, local governments have not exhibited a linear or uniform response. The interactive structure between policy adoption speed and the degree of text reproduction essentially represents a dynamic strategic game in which local governments navigate between institutional pressures and autonomous policy demands. In certain regions, rapid adoption with low variation represents the most direct response to central policies. This apparent loyal implementation is not entirely driven by policy endorsement, but rather by compliance with institutional assessments and regulatory requirements. When central evaluation mechanisms prioritize response speed as the main measure, local governments tend to replicate policies quickly to mitigate risks and ensure political safety. This phenomenon aligns with the logic of forced diffusion but also exposes a contraction in local governments' intentions for autonomous governance.

However, there are also regions where, despite rapid adoption, policy texts are significantly adjusted. This seemingly contradictory behavior is, in fact, a balance that local governments seek between institutional compliance and local interests. By quickly adapting within a limited timeframe, local governments not only respond to the speed pressure from higher authorities but also secure autonomous space for local development. This strategic choice reveals the operational logic of “legitimacy compliance” and “contextual innovation” in the policy diffusion process. Local governments are not passive implementers, but active strategic designers within the institutional framework.

Correspondingly, some local governments have adopted a deliberate delay strategy in policy adoption. Such delay does not necessarily indicate inactivity or negligence; rather, it represents a proactive effort to create an adjustment window and mitigate uncertainty risks (26). By postponing their responses, provincial governments gain opportunities to observe others' experiences and refine their own implementation paths. When delayed adoption is accompanied by high variation, it reflects the functioning of a learning-based diffusion mechanism, wherein local governments accumulate knowledge over time and optimize the local adaptability of policy execution through iterative adjustments (55). However, a distinct pattern of delayed adoption combined with low variation also emerges. This pattern reveals a structural issue in which local governments fail to utilize the observation period for meaningful policy reproduction, instead engaging in a combination of delayed execution and formalistic adoption. Such a “delay + minimal adjustment” model is more common in regions with limited resources and weak innovation capacity, reflecting the dilemma of passive diffusion and policy dependency.

Overall, local governments do not simply adopt policies faster or slower, nor merely modify or replicate texts; rather, they seek a relatively optimal governance path amid institutional constraints, resource conditions, and local development interests. The diffusion of sports industry policies thus exhibits a governance logic characterized by high strategic orientation and dynamic adaptability.

### Regional differences in policy text reproduction

The reproduction of sports industry policy texts across regions exhibits proximity, imitation, and clustering effects, reflecting the combined influence of institutional environments, resource endowments, and administrative inertia. This finding aligns with existing studies on geographical proximity effects, which show that neighboring provinces display a high degree of consistency in the extent of textual adjustments. This indicates that policy diffusion occurs not only through vertical transmission but also via horizontal learning and imitation (56). Regions in close geographical proximity, sharing similar socio-economic contexts and governance demands, tend to draw on each other's experiences, thereby forming convergent paths of policy adaptation (57).

Further regional comparisons reveal that the eastern provinces generally exhibit higher levels of textual variation. The advantages of economically developed regions—in terms of resources, governance capacity, and tolerance for policy innovation—enable local governments to flexibly adjust policy structures while responding to central directives, ensuring better alignment with local development needs. In these regions, deepened textual updates have become an important means to enhance policy implementation effectiveness and expand the scope of local autonomy.

In contrast, western and remote regions exhibit lower levels of text variation. Limited resources and stronger administrative inertia lead local governments in these areas to adopt more conservative strategies during policy adoption, favoring the direct application of central policy frameworks to minimize adjustment risks. This disparity indicates that institutional environments and resource allocations not only influence the speed of policy adoption but also profoundly shape the modes and depth of policy text reproduction.

Thus, the regional differences observed in the reproduction of sports industry policy texts are essentially the result of the interplay between institutional flexibility, local autonomy, and resource endowments. This finding suggests that understanding the relationship between central and local policy diffusion requires moving beyond traditional measures such as response speed or superficial adoption rates, and calls for a deeper analysis of how local institutional conditions systematically influence policy refinement and innovation behaviors.

### Theoretical contributions and practical implications

Based on the empirical case of sports industry policy diffusion, this study proposes a new perspective on the dynamic strategic choices made by local actors in the central-local policy transmission process, thus extending the theoretical understanding of local behavioral logics within policy diffusion research. Theoretically, the findings demonstrate that policy diffusion is not a unidirectional transmission of normative mandates from the center to the periphery, but a dynamic process characterized by continuous negotiation and mutual adjustment between central institutional arrangements and local agency. Rather than mechanically responding to central directives, local governments flexibly recalibrate policy texts and implementation pathways according to their resource endowments and developmental priorities during the processes of adoption and reproduction. This study shifts the focus of diffusion theory from merely the speed and scope of policy dissemination to the quality of diffusion and the depth of local adaptation, highlighting the constructive and proactive role of local governments within multi-level institutional architectures.

In practice, within China's national sports policy governance system, the findings of this study contribute to more effective monitoring and evaluation of policy implementation processes, further clarifying how local governments respond to higher-level authorities and providing empirical evidence for policy optimization. Specifically, central sports authorities can adopt the “variation coefficient of policy text reproduction” as a quantitative evaluation tool to regularly monitor the degree of deviation between provincial and central policies. This allows for the identification of “high-consistency regions” and “innovation-oriented regions”, providing decision-making references for policy supervision and best-practice dissemination. Similarly, provincial governments can use this metric to assess how well their policies align with national strategic objectives, enabling differentiated adjustments and targeted optimization in subsequent policymaking.

While the conclusions of this study offer comparative insights for other multilevel governance systems (e.g., the United States, Germany, and India), their applicability remains influenced by institutional structures and political-cultural contexts. Therefore, the “variation coefficient of policy text reproduction” should be viewed as a reference tool for analyzing policy implementation disparities across governance systems. Future research could further validate and compare this framework in diverse political contexts to enrich the cross-institutional understanding of policy diffusion mechanisms.

## Conclusion

Based on the perspective of policy diffusion, this study systematically analyzes the sports industry policy behaviours of China's 31 provincial governments from 2010 to 2023 within the process framework of “innovation cognition—policy adoption—policy text reproduction”. The aim is to enable the central government to better monitor the diffusion and implementation effectiveness of central sports industry policies and to provide a theoretical framework for provincial governments to improve their policy text reproduction practices.

This study explores the overall landscape of the relationships among policy adoption speed, adoption text volume, and the degree of variation in policy text reproduction across 31 provincial governments' sports industry policies. It introduces the “variation degree of policy text reproduction” as a new quantitative indicator, expanding policy diffusion research from the simple adoption behaviours of local governments to their policy text reproduction behaviours, revealing the autonomy and innovativeness of local governments in the policy diffusion process. The findings suggest that policy texts are not only tools for information transmission but also serve as vehicles for local governments to implement context-specific policy innovations. This indicates that the degree of variation in policy texts during transmission can reflect the flexibility with which local governments respond to central policies, providing a valuable addition to policy diffusion theory.

This study observes not only the “top-down” vertical mandatory diffusion behaviours but also instances of “bottom-up” reverse diffusion in policy experimentation (58). It proposes that “bottom-up” policy diffusion involves performance evaluation, incentive mechanisms between central and local governments, as well as innovation competition among peer governments (59). These phenomena indicate that, in the policy implementation process, local governments are not merely passive executors of central policies but also active participants and innovative architects. They aim to attract higher-level attention to specific policy alternatives, thereby forming a dynamic policy diffusion network.

The policy text reproduction behaviours of provincial governments are influenced by factors such as local development needs, regional geographic location, and the complexity of policy content. There is a significant cross-regional difference in variation coefficients, which increase gradually from the eastern coastal regions to the western inland areas. Economically developed provinces tend to make innovative adjustments, while remote provinces rely more heavily on the guidance of central policies. Geographically adjacent provinces are more likely to exhibit similar policy text reproduction behaviours.

Based on the characteristics of policy adoption speed and the degree of variation in policy text reproduction among provincial governments, this study identifies four typical response patterns: rapid adoption + low variation, rapid adoption + high variation, slow adoption + high variation, and slow adoption + low variation. The distribution patterns of these types suggest that policy diffusion is not a unidirectional transmission process, but rather the result of dynamic adjustments and strategic choices made by local governments between institutional constraints and local demands. This further reveals the diversity and complexity of the central-local policy diffusion mechanism.

## Limitations and future recommendations

This study faced data gaps due to limitations in the transparency and completeness of policy information from certain local governments, which affected the accurate assessment of policy adoption in some regions. Additionally, due to the lag in data collection, it was not possible to fully capture the latest developments in policy implementation. Future research should aim to improve data collection methods to ensure the timeliness and completeness of data, thereby enhancing the accuracy and reliability of the analysis results.

This study is based solely on data from 31 provinces in China. Although it covers the national scope, it primarily focuses on China's domestic policy system and does not fully account for variations in an international context. Differences in policy environments, levels of economic development, and cultural backgrounds across countries and regions may influence policy adoption behaviours and implementation outcomes. Therefore, future research could broaden its scope to an international level, incorporating policy adoption cases from other countries and regions for comparative analysis. This would enhance the global applicability of the conclusions and provide a broader reference for global policymaking.

This study is primarily based on the analysis of policy texts, which serve as a concentrated expression of institutional intentions and can accurately reflect governments' cognitive orientations and action frameworks in the processes of policy design and adjustment. However, policy adoption and text reproduction represent only one stage in the overall policy implementation process. Future research could build upon this foundation by integrating data on policy performance evaluation, resource allocation, and social feedback, thereby deepening the understanding of the diffusion mechanisms of sports industry policies from the perspectives of implementation pathways and practical outcomes.
